# Structured pelvic floor physiotherapy rehabilitation for low anterior resection syndrome in colorectal cancer: An Australian feasibility study

**DOI:** 10.1007/s00520-026-10777-w

**Published:** 2026-05-27

**Authors:** Kin Yin Carol Chan, Michael Suen, Gemma Collett, Susan Coulson, Janindra Warusavitarne, Janette L. Vardy

**Affiliations:** 1 Concord Cancer Centre, Concord Repatriation General Hospital, Sydney, Australia; 2 Faculty of Medicine and Health, University of Sydney, Sydney, Australia; 3 Department of Colorectal Surgery, Concord Repatriation General Hospital, Sydney, Australia; 4 Mark’s Hospital, London North West, England; 5 Australian Research Centre for Cancer Survivorship, University of New South Wales, Sydney, Australia

**Keywords:** Low anterior resection syndrome, Pelvic floor rehabilitation, Colorectal cancer survivorship, Bowel function, Physiotherapy

## Abstract

**Purpose:**

Low Anterior Resection Syndrome (LARS) is a common and debilitating outcome of sphincter-preserving surgery for colorectal cancer, severely affecting quality of life. While pelvic floor rehabilitation (PFR) is recommended as a conservative treatment, access to structured care is limited. This study assessed the feasibility and acceptability of a structured, physiotherapist-led PFR programme in an Australian outpatient setting, and explored the within-person changes in bowel function and quality of life.

**Methods:**

A non-randomised, single-arm prospective study was conducted at Concord Repatriation General Hospital (Sydney, Australia) from September 2020 to April 2024. Colorectal cancer survivors with LARS (score > 20) and bowel continuity restored > 6 months previously were enrolled. The 10-week PFR programme included education, pelvic floor muscle training, rectal balloon biofeedback, and home exercises, with adaptations for telehealth due to COVID-19. Primary outcome was programme adherence. Secondary outcomes included bowel, bladder, and sexual function, quality of life, and anorectal physiology; measured at baseline, post-intervention (3 months) and follow-up (9 months).

**Results:**

Fourteen participants (median age sixty-three; seven female) completed the programme (one dropout with non-clinical reason), with 100% attendance and high home exercise adherence (median completion 100%). Bowel function improved significantly (median LARS score reduction -13.0; *p* = 0.004), with 71.4% achieving meaningful change post-intervention and 63.6% at follow-up. Quality of life significantly improved on validated measures. Anorectal physiology showed increased anal pressures, sensory thresholds, and better defaecatory coordination. No adverse events were reported.

**Conclusions:**

A structured, physiotherapist-led PFR programme is feasible and acceptable for colorectal cancer survivors with LARS. While improvements in bowel function and quality of life were observed over time, these findings should be interpreted as exploratory. The hypothesis-generating findings support further evaluation of PFR in a controlled trial to evaluate effectiveness and inform integration into multidisciplinary survivorship care.

## Introduction

Low Anterior Resection Syndrome (LARS) is a common and often debilitating functional consequence of sphincter-preserving surgery for colorectal cancer (CRC) [1, 2]. It is characterised by a constellation of symptoms, including faecal urgency, increased frequency, incontinence, and difficulties with evacuation [3–5]. The severity of LARS varies; however, patients who undergo temporary stoma formation, radiotherapy, or low anastomosis are at a greater risk of developing symptoms [6]. LARS affects up to 80% of CRC survivors, with 38–62% meeting criteria for major LARS [7–9]. In regional Australian settings, ~ 55% of patients report LARS, with 37.5% experiencing major LARS [10]. LARS symptoms impair physical function, psychosocial well-being and overall quality-of-life (QOL) [11–13]. 

Various treatment options for LARS management include pharmacological methods, conservative management, and surgical intervention [14, 15]. Optimised conservative management (OCM) is recommended as first-line management and involves lifestyle and behavioural modifications, dietary adjustments, and pelvic floor rehabilitation (PFR) [16, 17]. PFR offers a structured, patient-centred approach through pelvic floor muscle training (PFMT) and biofeedback (BF) aiming to re-establish anorectal coordination and rectal sensory discrimination and restore pelvic floor muscle function [18–20]. A randomised controlled study demonstrated PFMT initiated early in postoperative recovery was associated with a lower LARS score compared with standard care [19]. PFR in conjunction with biofeedback, education and lifestyle advice has been associated with improved QOL [21]. These findings suggest PFR may confer meaningful patient-reported benefits; however, variability in intervention protocols and care settings limits generalisability.

Despite the growing recognition of LARS and emerging international evidence supporting PFR, a standardised protocol for PFR is lacking, and access to coordinated care remains limited in Australia [20, 22, 23]. Many patients receive fragmented, reactive care instead of proactive, structured rehabilitation. Evidence regarding the feasibility and implementation of PFR programmes in the Australian healthcare system is scarce, particularly given differences in service delivery models across Europe and North America. Major gaps include unclear referral pathways, inadequate integration with oncology and surgical services, and lack of tailored care to address individual patient needs, highlighting the need for localised evidence to inform service feasibility and implementation [24, 25]. This study addresses this gap by evaluating the feasibility and acceptability of a structured, physiotherapist-led PFR programme delivered in an Australian outpatient CRC setting, while exploring within-person changes in bowel function and QOL.

## Methods

### Study design

This is a non-randomised, single-arm, prospective study evaluating the feasibility of a physiotherapist-led PFR in CRC patients with LARS after sphincter-preserving anterior resection. Recruitment occurred at Concord Repatriation General Hospital (CRGH), a tertiary hospital in Sydney, Australia, from September 2020 to April 2024. The recruitment period was extended due to the COVID-19 pandemic, and the study protocol was adapted to include a telehealth component.

### Participants

Patients attending Sydney Cancer Survivorship Clinic, Colorectal Surgery Outpatient Clinics at CRGH, or the clinics of colorectal surgeons in New South Wales, Australia, were invited to participate. Eligibility criteria included: (i) anterior resection with sphincter preservation for CRC ± neoadjuvant/adjuvant therapy; (ii) onset of bowel dysfunction symptoms post-surgery and treatment, with a LARS Score > 20; (iii) bowel continuity restored for ≥ 6 months; and (iv) no evidence of recurrent CRC or distant metastasis. Assessment at six months was selected, making it an ideal time for prompt intervention [26]. Exclusion criteria included: (i) age under 18; (ii) inability to give informed consent or follow instructions due to cognitive or English language difficulties; (iii) neurological disorders or acute exacerbation of inflammatory bowel disease. Written consent was obtained before enrolment.

### PFR Intervention

PFR intervention was delivered by a single physiotherapist trained in pelvic floor dysfunction and rectal balloon biofeedback. Attendance and home exercise compliance were monitored weekly. PFR began with assessments, including Patient-Reported Outcome Measures (PROMS), a clinical examination, and collection of clinical data, followed by ten weekly 60-min sessions. Originally conducted in person, the programme adapted to telehealth in July 2021 due to COVID-19 restrictions, offering supervised PFM training via secure videoconferencing.

The first session included education on pelvic floor and anorectal anatomy, LARS post-CRC surgery, symptom management strategies (including diet, optimal toileting, and bowel/bladder habits), and correct pelvic floor muscle (PFM) activation. Educational handouts were provided. The ten-week programme focused on anorectal coordination, pelvic floor muscle (PFM) training, and overall functional performance. It involved three key components: 1) Anorectal Coordination with Biofeedback using a rectal balloon catheter to elicit sensations of rectal distension, urge, and maximal volume, with instructions to activate pelvic floor muscles and expel the balloon at maximal volume. 2) PFM Training, including visual feedback with a transperineal ultrasound [27, 28] to ensure proper muscle activation, followed by structured exercises for strength, endurance, and reaction with a focus on relaxation and breathing. The training also integrated functional exercises across various positions and used resistance equipment. 3) Home Exercise Programme, including a bowel symptom diary to record exercises and bowel symptoms daily. Diaries were reviewed weekly (in person or via telehealth) to monitor progress and guide further exercise prescription.

### Outcomes

Outcome measures were selected to evaluate feasibility, acceptability, and preliminary clinical signals of benefit associated with the PFR programme. Data were collected at three time-points: baseline (0-month), post-intervention (3-months from baseline), and follow-up (9-months from baseline/6-months post-intervention), with the first two assessments conducted in person and the final one via phone interview (PROMS only).

The primary endpoint was feasibility, including adherence and completion. Adherence was assessed through therapist-recorded attendance at supervised sessions, completion of exercise diaries and exercise frequency. Participant satisfaction and acceptability were assessed at program completion using an investigator-developed survey.

### Clinical outcomes

Secondary endpoints to explore within-person changes over time were measured by validated questionnaires. Bowel function was assessed using the Low Anterior Resection Syndrome Score (LARS) and Memorial Sloan Kettering Cancer Centre–Bowel Function Instrument (MSKCC-BFI) which capture symptom severity and functional impact of bowel dysfunction [29, 30]. Bladder function (International Consultation on Incontinence Questionnaire–Lower Urinary Tract Symptoms [ICIQ-LUT]) [31], and sexual function (International Index of Erectile Function [IIEF], Female Sexual Function Index [FSFI]) [32, 33] were also evaluated. QOL was assessed using condition-specific and cancer-specific instruments [34–36] (Faecal Incontinence Quality of Life [FIQOL], Hospital Anxiety and Depression Scale [HADS], Functional Assessment of Cancer Therapy–Colorectal [FACT-C]). PROMS were self-administered by participants at each assessment time-point.

Objective anorectal function, using anorectal physiology assessment, was conducted at baseline and post-intervention (3-months) to explore physiological correlates of symptom change, using high-resolution anorectal manometry, 24-channel water-perfused manometry catheter and endoanal ultrasound (Hitachi Aloka medical diagnostic ultrasound system).

### Feasibility justification

The sample size was pragmatic, based on the number of eligible CRC patients attending CRGH outpatient clinics and the number of anterior resections performed over the previous two years. Given that 30% were likely to report LARS, the aim was to recruit 15 participants over a 24-month period.

### Statistical analysis

Statistical analyses were conducted using IBM SPSS Statistics 26.0. Feasibility outcomes included adherence, completion and compliance with PFR program. Attendance was recorded by the treating-therapist and defined as the proportion of scheduled therapy sessions attended by each participant. Exercise adherence was assessed using home exercise diaries, in which participants recorded the frequency and completion of prescribed home exercises (once daily, at the prescribed intensity and repetitions). Exercise adherence was calculated as the proportion of prescribed exercise sessions over the study period, based on the diary records reviewed by the physiotherapist at each session, including during telehealth consultations. Program completion was defined as attendance at ≥ 80% of scheduled sessions. Overall compliance was defined as meeting ≥ 80% of both attendance and home exercise adherence requirements. An investigator-developed satisfaction survey was administered at program completion to assess overall satisfaction with the program. Demographic characteristics were reported using descriptive statistics. Given the small sample size and non-normal distribution of repeated-measures data, non-parametric tests were applied. Changes in LARS, MSKCC-BFI, FIQoL and FACT-C scores across three time-points (baseline, post-intervention and follow-up) were analysed using the Friedman test. Where significant overall effects were found, Conover post hoc tests with Bonferroni correction were used for pairwise comparisons. Effect sizes were calculated using Kendall’s W for overall effects and rank-biserial correlation (rrb) for pairwise comparisons, with values interpreted as small (± 0.1), moderate (± 0.3), and large (± 0.5) effects; the sign of the coefficient indicates the direction of the association. Statistical significance was set at *p* < 0.05. Missing data were handled using a single imputation method, in which the group median replaced missing values in continuous variables.

## Results

**Fig. 1:**
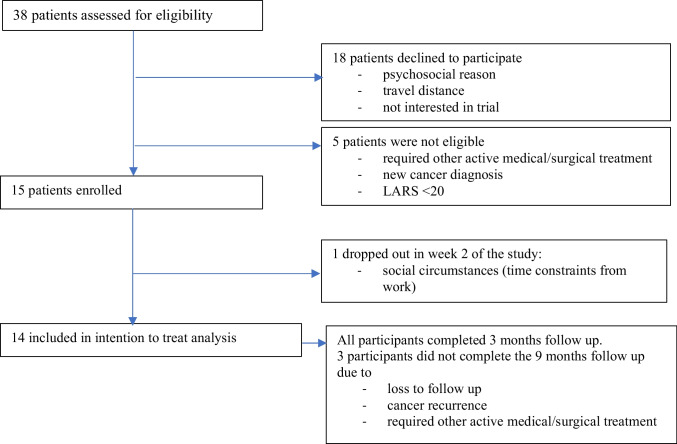
Study Recruitment Fifteen patients enrolled. Fourteen included in intention-to-treat analysis

Participant recruitment opened in September 2020 but due to COVID-19 restrictions, the first enrolment occurred in May 2021. Of thirty-eight patients assessed for eligibility, twenty-three did not enrol. Reasons for non-enrolment included lack of interest, competing work or personal commitment, health-related issues, logistical barriers to attendance, COVID-19-related concerns, and loss to follow-up. Three patients were excluded due to a LARS score < 20, and two were deemed not clinically suitable for study enrolment (Table 1). The targeted sample size of fifteen participants was achieved in January 2024. Figure 1 provides an overview of study recruitment. Baseline characteristics are shown in Table 2. One patient withdrew in week 2 due to social circumstances (time commitment due to work). This participant was included in the baseline characteristics and partial adherence data. Fourteen participants completed the programme and were included in the final comparative analysis. One participant did not return the home exercise diary, and one did not complete the post-intervention anorectal physiology study. At follow-up (9 months from baseline), outcome data were unavailable for three participants due to: lost to follow-up (*n* = 1), cancer recurrence (*n* = 1), and attending other active medical treatment (*n* = 1). No adverse events were reported.

**Table 1:** Reasons for non-enrolment

Recruitment flow category	Reason for non-enrolment	*n*
Declined to participate (*n* = 18)	Not interested/declined participation	6
	Unable to commit due to work or other commitments	5
	Lost to follow-up/unable to contact	3
	Health-related reasons (received other medical advice)	2
	COVID-19 related concerns	1
	Logistical barriers (distance to travel)	1
Not eligible (*n* = 5)	Required other active medical/surgical treatment	1
	New diagnosis of other cancer type	1
	Did not meet LARS eligibility criteria after screening (LARS score < 20)	3

**Table 2:** Baseline Characteristics (*n* = 15)

Demographics	
Age [median (IQR)] (year)	63 (52.5–68.5)
Gender [*n* (%)]	
Male	8 (53.5)
Female	7 (46.7)
BMI [median (IQR)] (kg/m^2^)	26.0 (24.1–28.7)
Childbirth history [*n* (%)]	
Female	7 (100)
Smoking [n (%)]	
Never	8 (53.3)
Previous	6 (40)
Current	1 (6.7)
Marital status [*n* (%)]	
Married	10 (66.7)
Divorced	2 (13.3)
Widow	3 (20)
Employment status [*n* (%)]	
Unemployed	1 (6.7)
Self- employed	4 (26.7)
Employed full time	3 (20)
Retired	7 (46.7)
Functional Status—Mobility	
Independent	15 (100)
Dependent	0 (0)
Functional Status – Activities of Daily Living	
Independent	15 (100)
Dependent	0 (0)
Other medical history [*n* (%)]	
Hypertension	6 (40)
Diabetes Mellitus	1 (6.7)
Both	1 (6.7)
None	7 (46.7)
Surgical information	
Cancer stage [*n* (%)]	
Stage I	3 (20)
Stage II	5 (33.3)
Stage III	7 (46.7)
Cancer location [*n* (%)]	
Sigmoid	2 (13.3)
Rectum	13 (86.7)
Operative approach [*n* (%)]	
Open	1 (6.7)
Laparoscopic	10 (66.7)
Hand Assisted Laparoscopic	3 (20)
Robotic	1 (6.7)
Type of surgical procedure [*n* (%)]	
High Anterior Resection	1 (6.7)
Low Anterior Resection	3 (20)
Ultra-low Anterior Resection	11 (73.3)
Total Mesorectal Excision [*n* (%)]	
Partial mesorectal excision	1 (6.7)
Total mesorectal excision	14 (93.3)
Anastomotic height from anal verge [*n* (%)]	
≥ 11 cm	1 (6.7)
6–10.9 cm	3 (20)
0–5.9 cm	11 (73.3)
Type of Reconstruction [*n* (%)]	
End to end anastomosis	10 (66.7)
Side to end anastomosis	3 (20)
Colonic J pouch anastomosis	1 (6.7)
Coloanal anastomosis	1 (6.7)
Surgical complications [*n* (%)] (UTI, SBO, Anastomotic leak × 2, Adhesiolysis)	
Yes	6 (40)
Anastomotic leak	2
SBO	2
UTI	2
No	9 (60)
Stoma [*n* (%)]	
Yes	11 (73.3)
No	4 (26.7)
Stoma duration [median (IQR)] (months)	4 (2.5–7.5)
Time since bowel continuity restored [median (IQR)] (months)	18.0 (8–24)
Oncological treatment	
Neoadjuvant therapy [*n* (%)]	
Yes	5 (33.3)
No	10 (66.7)
Neoadjuvant therapy type [*n* (%)]	
Long course neoadjuvant chemoradiotherapy	4 (26.7)
Short course neoadjuvant radiotherapy	1 (6.7)
None	10 (66.7)
Adjuvant therapy [*n* (%)]	
Yes	7 (46.7)
Fluorouracil + Oxaliplatin + Folinic acid	1
Capecitabine + Oxaliplatin	4
Capecitabine	1
Missing	1
No	8 (53.3)

### Adherence and Satisfaction

The programme had a 94.7% attendance when including all 15 participants. Excluding the participant who withdrew, attendance was 100% with all remaining participants meeting the predefined completion criterion of attending > 80% of scheduled sessions. Home exercise adherence was high, with a median completion of 100% (IQR 92–100%) among participants who returned exercise diaries (13/14).

Due to COVID-19 restrictions, 8/14 participants completed the program using a hybrid delivery model (adapted protocol). The number of telehealth sessions delivered to the participants ranged from 1 to 6 sessions. Quantitative satisfaction outcomes indicated high acceptability of the program, with 60% rating it “excellent”, 26.7% “very good” and 6.7% “good”. In-depth satisfaction outcomes, including qualitative findings from exit interviews, will be reported elsewhere.

### Bowel function and quality of life

#### Minimal Clinically Important Difference (MCID) for LARS

The established Minimal Clinically Important Difference (MCID) for the LARS score is 5 points [37]. The median change in LARS scores from baseline to post-intervention was −13 (95% CI: −20.0 to −6.5, *p* = 0.004). At post-intervention (*n* = 14), 10/14 participants (71.4%) exhibited a clinically meaningful improvement. Overall, 7/11 (63.6%) participants who completed the follow-up (*n* = 11) showed a clinically meaningful within-person improvement.

#### LARS Category

**Fig. 2:**
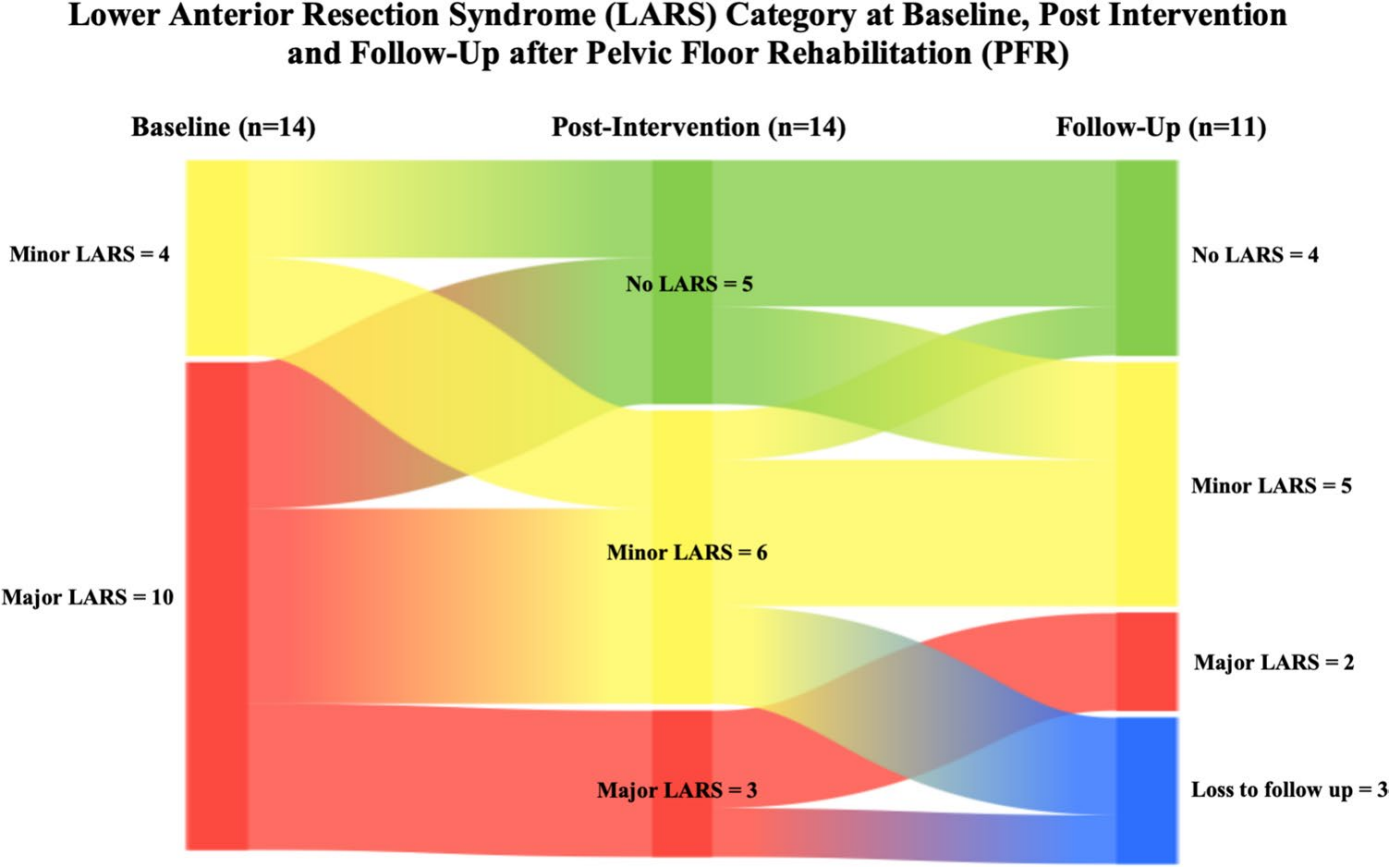
Lower Anterior Resection Syndrome (LARS) category at baseline, post-intervention and follow-up after Pelvic Floor Rehabilitation (PFR)

At baseline, 10/14 participants (71.4%) were classified as experiencing major LARS. At post-intervention, this decreased to 3/14 participants (21.4%), with five participants classified as having no LARS (35.7%). In total, 9/14 (64.2%) participants showed improvement by at least one LARS category: three participants moved from major LARS to no LARS, four from major to minor LARS, and two from minor to no LARS. At follow-up, 6/11 participants showed sustained improvement in LARS categories. One participant demonstrated further improvement, transitioning from minor LARS to no LARS (Fig. 2).

#### LARS score and MSKCC bowel function instrument

The median LARS Score decreased from baseline (*n* = 14) (M = 36.0, IQR 30.0–39.0) to post-intervention (*n* = 14) (M = 23.0, IQR 15.3–29.0) and remained improved at follow-up (*n* = 11) (M = 26.0, IQR 20.0–27.5). A Friedman test revealed a significant change in LARS scores across the three time points χ^2^(2) = 12.67, *p* = 0.002, with a large effect size (Kendall’s W = 0.576). Post hoc Conover tests showed significant improvements from baseline to post-intervention (*p* < 0.001, rrb = 0.945) and from baseline to follow-up (*p* = 0.002, rrb = 1.000). No change was observed between post-intervention and follow-up (*p* = 1.000), indicating gains were maintained.

Participants demonstrated associated improvements in bowel function measured by the MSKCC-BFI, with total median score increased from 58.0 at baseline to 68.0 post-intervention, an overall improvement of + 9.0 points (95%CI 4.0, 14.0, *p* = 0.004). Improvements were observed across all MSKCC subdomains, including frequency (+ 4.5), urgency (+ 5.4), soilage (+ 1.6), and diet (+ 0.21), suggesting broad-based functional gains. Similarly, MSKCC-BFI scores showed a significant overall difference over time, χ^2^(2) = 8.00, *p* = 0.018, with a moderate effect size (Kendall’s W = 0.333). Post hoc comparisons revealed significant improvements from baseline to both post-intervention and follow-up (*p* = 0.027 for both), with large effect sizes (rrb = –0.859 and –0.872, respectively). No difference was found between post-intervention and follow-up (*p* = 1.000), suggesting sustained benefit (Table 3). At follow-up, outcome data were missing for three participants; missing continuous outcome values were imputed using the group median as described in the Statistical Analysis section.

**Table 3:** Changes in functional and QOL scores across time points

Measure	Baseline, median (IQR)	Post-Intervention, median (IQR)	Follow-Up, median (IQR)	Friedman χ^2^(df)	p	Kendall’s W	Comparison	P _bonf_	Rank-Biserial Correlation (rrb)
LARS	36.0 (30.0–39.0)	23.0 (15.3–29.0)	26.0 (20–27.5)	12.67 (2)	0.002	0.576	Baseline vs Post-Intervention	< 0.001	0.945
							Baseline vs Follow-Up	0.002	1.000
							Post-Intervention vs Follow-Up	1.000	–0.556
MSKCC-BFI	58.0 (48.5–63.8)	68.0 (55.5–72.0)	68.0 (63.0–71.0)	8.91 (2)	0.012	0.405	Baseline vs Post-Intervention	0.008	–0.909
							Baseline vs Follow-Up	0.027	–0.848
							Post-Intervention vs Follow-Up	1.000	0.439
FIQoL	2.3 (1.7–3.2)	3.4 (2.5–3.8)	3.5 (3.1–4.0)	14.73 (2)	< 0.001	0.669	Baseline vs Post-Intervention	0.014	–0.848
							Baseline vs Follow-Up	< 0.001	–0.909
							Post-Intervention vs Follow-Up	0.014	–0.667
FACT-C	101.5 (84.3–117.0)	112.8 (95.8–125.0)	114.0 (106–129)	8.91 (2)	0.012	0.405	Baseline vs Post-Intervention	0.008	–0.788
							Baseline vs Follow-Up	0.027	–0.682
							Post-Intervention vs Follow-Up	1.000	–0.045
ICIQ-MLUT	10.0 (3.5–10.5)	6.0 (3.5–8.5)	6.0 (3.0–9.0)	3.00 (2)	0.223	0.214	Baseline vs Post-Intervention	0.651	0.667
							Baseline vs Follow-Up	0.323	0.500
							Post-Intervention vs Follow-Up	1.000	–0.048
IIEF	47.5 (31.3–60.3)	62.5 (48.8–67.8)	57.0 (44.5–60.3)	4.43 (2)	0.109	0.554	Baseline vs Post-Intervention	0.412	–1.000
							Baseline vs Follow-Up	1.000	0.200
							Post-Intervention vs Follow-Up	0.108	1.000
ICIQ-FLUT	13.0 (7.0–15.5)	7.0 (5.5–8.0)	4.5 (1.8–8.8)	2.80 (2)	0.247	0.350	Baseline vs Post-Intervention	0.852	1.000
							Baseline vs Follow-Up	0.384	0.800
							Post-Intervention vs Follow-Up	1.000	0.200

*LARS* Low Anterior Resection Syndrome, *MSKCC-BFI* Memorial Sloan Kettering Cancer Centre- Bowel Function Instrument, *FIQOL* Faecal Incontinence Quality of Life, *FACT-C* Functional Assessment of Cancer Therapy- Colorectal, *ICIQ-MLUT* International Consultation on Incontinence Modular Questionnaire- Male Lower Urinary Tract Symptoms, *IIEF* International Index of Erectile Function, *ICIQ-FLUT* International Consultation on Incontinence Modular Questionnaire- Female Lower Urinary Tract Symptoms. FSFI was not included due to all female participants not being sexually active

#### Quality of life (FIQOL and FACT-C)

Both instruments showed significant associated improvement after the intervention. For FIQL, there was a significant overall change over time (Friedman χ^2^(2) = 14.73, *p* < 0.001; Kendall’s W = 0.669), with post‑hoc tests indicating improvement from baseline (*n* = 14) to post‑intervention (*n* = 14) and to follow‑up (*n* = 11), and further gains from post‑intervention to follow‑up (all *p* ≤ 0.014; large effects). For FACT‑C, overall improvement was observed (Friedman χ^2^(2) = 8.91, *p* = 0.012; W = 0.405), with increases from baseline to post‑intervention and to follow-up (*p* = 0.008 and 0.027, respectively) (Table 3). At follow-up, outcome data were missing for three participants; missing continuous outcome values were imputed using the group median as described above.

#### Anorectal physiology assessment

Anorectal physiology testing (*n* = 13) demonstrated improvement in several key parameters following the intervention. Resting anal pressure increased from 39.4 mmHg (IQR 29.0–62.8) to 51.1 mmHg (IQR 38.7–65.1), but was not statistically significant (median difference 7.6 mmHg, 95% CI − 1.9 to 17.2; *p* = 0.127). Squeeze pressure improved by 15.6 mmHg (95% CI 0.55 to 28.8; z = 2.062, *p* = 0.04), suggesting improvement in voluntary sphincter contraction. Rectal sensory thresholds increased for desire to defaecate (HL + 25 mL, 95% CI + 10 to + 40; z = 2.667, *p* = 0.008) and maximum tolerated volume (HL + 30 mL, 95% CI + 20 to + 45; z = 2.934, *p* = 0.004), whereas changes in first sensation volume were not statistically significant (*p* = 0.374). Simulated defaecation relaxation improved from 8.0% to 25.0% (HL + 18%, 95% CI + 4.5% to + 30.5%; z = 2.516, *p* = 0.013), suggesting improved defaecatory coordination (Table 4). One participant with missing outcome data was excluded from the relevant analyses, and no imputation was performed.

**Table 4:** Paired Sample t-tests of anorectal physiology parameters and stool diary comparing baseline and post-intervention

Outcome Measure (Post intervention – Baseline)	Baseline, median (IQR)		Post-Intervention, median (IQR)	z		p	HL Median Paired Difference (95% CI)	Rank‑biserial *r* (95% CI)
Anorectal Physiology								
Resting Pressure (mmHg)	39.4 (29.0–62.8)	51.1 (38.7–65.1)		1.572	0.127		+ 7.6 (− 1.85 to + 17.15)	0.50 (− 0.07 to 0.82)
Squeeze Pressure, mean (mmHg)	136.8 (82.2–170.1)	144.1 (96.5–209.8)		2.062	0.040		+ 15.55 (+ 0.55 to + 28.75)	0.65 (0.16 to 0.88)
Endurance (sec)	131.2 (85.1–136.2)	142.8 (89.3–155.8)		1.223	0.244		+ 5.7 (− 5.35 to + 15.4)	0.39 (− 0.21 to 0.77)
Simulated Defaecation Relaxation (%)	8.0 (3.0–9.0)	25.0 (16.0–29.0)		2.516	0.013		+ 18.0 (+ 4.5 to + 30.5)	0.79 (0.43 to 0.93)
First sensation volume (ml)	30 (20–40)	30 (20–40)		0.917	0.374		+ 5 (− 10 to + 20)	0.33 (− 0.35 to 0.78)
Desire to defaecate volume (ml)	40 (30–50)	50 (50–90)		2.667	0.008		+ 25 (+ 10 to + 40)	0.91 (0.69 to 0.98)
Maximum tolerated volume (ml)	40 (30–70)	60 (50–100)		2.934	0.004		+ 30 (+ 20 to + 45)	1.00 (1.00 to 1.00)
Stool Diary								
Number of Anal Incontinence episodes per day1.0 (0.0–3.0)		0.0 (0.0–0.0)		−2.366	0.022		− 3.247 (− 6.0 to − 1.5)	− 1.00 (− 1.00 to − 1.00)
Bowel Frequency per day	4.0 (3.1–5.1)	3.3 (2.3–4.3)		−3.110	0.002		− 0.860 (− 1.405 to − 0.475)	− 0.98 (− 0.99 to − 0.93)

Wilcoxon signed rank test

#### Other functional measures

Anal incontinence and bowel frequency showed statistical improvement (Table 4). Other outcome measures (male and female bladder function, sexual function, Hospital Anxiety and Depression Scale, and Endoanal ultrasound) did not show significant differences.

## Discussion

This study supports the feasibility of delivering a structured, outpatient PFR programme for CRC survivors with LARS, demonstrating high adherence, completion and acceptability. Exploratory within-participant improvements in LARS symptoms and QOL were observed; however, given the single-arm design, these clinical findings should be interpreted as hypothesis-generating rather than confirmatory of clinical efficacy.

### Feasibility and acceptability of PFR

Our primary objective was to evaluate feasibility, and several indicators support that the delivery is safe and well-received by participants. High adherence to both supervised sessions and home exercises, as documented through therapist-recorded attendance and completed exercises, supports the feasibility of delivering a structured PFR program in the CRC population. Diary review during weekly sessions facilitated individuals’ motivation and technique reinforcement, which may have contributed to sustained engagement. These findings suggest that structured self-monitoring is an acceptable strategy to support adherence in the cancer rehabilitation model. Participants’ satisfaction was high, likely due to the perceived relevance, supportiveness, and empowering nature of the program, suggesting the value of PFR in CRC care [38]. However, long-term program sustainability should be considered in relation to its costs and implementation within a complex healthcare system. While the adherence rate in our study was high, we acknowledge this may vary across different healthcare systems due to resource and accessibility constraints, including the availability of trained staff, regional geographic considerations, and support for minority groups [39–41].

The incorporation of telehealth during COVID-19 disruptions further enhances feasibility, allowing continuity of care when in-person sessions were not possible. This modality provided flexibility without compromising adherence or completion. This approach aligns with broader evidence showing its effectiveness in managing pelvic floor dysfunction symptoms and supporting skill acquisition and retraining through physiotherapy [42, 43]. However, broader implementation may be limited for individuals with low digital literacy, language barriers, in areas with limited internet access, and rehabilitation training elements that require in-person attendance such as rectal biofeedback [44–46].

### Signals of clinical benefit

Exploratory analyses suggested improvements in LARS severity and QOL following PFR, as observed by patient-reported outcome measures (PROMS) and anorectal physiology (ARP) assessments. Over 70% of participants demonstrated clinically meaningful within-person improvements in LARS scores post-intervention, with 64% maintaining benefits at follow-up. Improvements in PROMS were observed immediately post-intervention and were generally maintained over time. This occurred alongside favourable changes in ARP measures, including sphincter squeeze pressures, rectal sensation, and defaecatory coordination, which are relevant to urgency, frequency, and incontinence. The parallel changes in PROMS and ARP suggest a mechanistic link between neuromuscular function and symptom resolution. Thus, convergence of subjective and objective measures supports the physiological plausibility of the intervention. However, given the study design, these associations should be interpreted as supportive rather than confirmatory evidence of the mechanism.

PFR is hypothesised to address the multifactorial nature of LARS, by improving pelvic floor and external anal sphincter muscle function, restoring anorectal coordination and optimising the responses to rectal filling, supporting both evacuation and storage mechanisms [17, 18, 47]. This multimodal PFR extended beyond PFM training with exercise and biofeedback, and incorporated education and behavioural strategies including bowel and bladder habits, optimal toileting strategies and dietary advice. These components are intended to reduce maladaptive behaviours such as excessive straining, incomplete emptying, to optimise stool consistency and minimise urgency-triggering behaviours, thereby complementing neuromuscular retraining [48–50]. The integration of biofeedback, education and behavioural strategies may facilitate motor relearning and improve awareness of anorectal function, reduce urgency-related anxiety and support the re-establishment of effective toileting patterns.

This study contributes to the growing body of evidence [51, 52] of improved LARS symptoms, as measured by PROMS and ARP, following PFR, while demonstrating the acceptability of a standardised, multimodal PFR approach. PROMS capture patients’ experiences and functional impact of symptoms on QOL [30, 34, 53]. When paired with ARP data, which offers an objective measure of bowel function, it provides a more comprehensive understanding of bowel function. The dual assessment approach also informs the design and implementation of individualised rehabilitation programmes tailored to specific symptoms and functional needs [54–57]. It offers a comprehensive evaluation of feasibility and potential treatment response, although its implementation may present challenges in resource-limited or rural settings [21]. Variation in individual response observed in this study highlights the need for future controlled trials to examine factors influencing treatment responsiveness, such as the chronicity of LARS, baseline function, comorbidities, psychosocial readiness for behaviour change, durability of benefit and long-term outcomes.

### Positioning of PFR within optimised conservative management

This study supports the potential role of PFR within the framework of LARS Optimised Conservative Management (OCM). Current stepwise approaches, such as the MANUEL and BOREAL protocols [20, 58], emphasise conservative treatment but lack standardised integration of structured, multidisciplinary PFR in CRC management. While prior studies by Asnong [19] and Van der Heijden [21] demonstrated benefits of PFR, heterogeneity in the intervention and outcome assessment limits comparability and implementation [59]. This includes establishing clear definitions of treatment duration, delivery methods, and outcome assessments. The recent POLARIS RCT protocol compared optimised conservative management with transanal irrigation and sacral neuromodulation [37]. However, their definition of OCM did not include structured and supervised PFR, likely due to resource limitations or disparities in standardised service access across trial centres. This omission raises important concerns about the standardisation of OCM and need for integrating PFR into the LARS management pathway.

The feasibility demonstrated in this study suggests that supervised, physiotherapist-led PFR could be incorporated consistently within CRC survivorship. However, its successful integration into standard care requires addressing workforce capacity, equitable access, and system readiness. Adherence to treatment protocols and appropriate timing will be essential for achieving meaningful outcomes [60]. Barriers such as a shortage of sufficiently trained physiotherapists and a lack of structured referral systems impede the widespread implementation of PFR in healthcare settings [61]. In Australia, there are no formal rehabilitation models for LARS in contrast to some European countries, where dedicated LARS clinics and structured referral systems are integrated into CRC care [59, 62, 63]. Currently, access to rehabilitation services is often based on ad hoc clinician referrals, which rely on individual clinician awareness rather than systematic integration into the care pathway. This fragmented approach can delay care, hinder interdisciplinary collaboration, and contribute to patient disengagement, especially when services are not incorporated into the multidisciplinary cancer care pathways [15]. Addressing these system-level barriers will be essential to scaling PFR beyond research settings.

### Strengths

The strengths of this study are its real-world relevance. The intervention consisted of a structured, multimodal rehabilitation program designed to actively engage patients in pelvic floor strengthening, coordination, and functional retraining. This behavioural and functional approach aimed to empower patients and promote sustainable self-management of LARS symptoms. While earlier intervention may benefit selected patients, the six-month eligibility criteria provide a practical balance between early identification of persistent LARS and feasibility within routine postoperative recovery pathways [4, 26]. The study included patients with minor and major symptoms, regardless of surgical approach [64]. There was no upper time limit on LARS chronicity, increasing applicability of the findings to both recent and long-standing cases.

### Limitations and future research

Recruitment was affected by the COVID-19 pandemic, with clinic closures, delays for elective surgeries and restrictions on face-to-face care, which all contributed to a small sample size. Although reasons for non-participation were documented at screening, more in-depth qualitative exploration of barriers to enrolment was not undertaken. Patient reluctance to engage in the trial possibly stemmed from stigma around bowel dysfunction, personal commitments, and a desire to avoid healthcare facilities and commuting due to COVID-19 risk. Consequently, important psychosocial, logistical and health system factors influencing trial participation may not have been fully captured. This might limit the interpretation of recruitment feasibility and generalisability. Formal frailty measures were not collected, although we did collect comorbidity and performance status. Future studies should consider incorporating a validated frailty scale to evaluate feasibility and scalability across a more functionally dependent population. All participants were recruited from a single tertiary centre and its affiliated clinic, which may limit external validity due to the potential bias and participant motivation.

The single-arm design, without a control group, precludes causal inference regarding the effectiveness of the intervention. Improvements observed over time may have been influenced by regression to the mean, or non-specific effects such as increased clinical attention. Results should be interpreted as hypothesis-generating rather than confirmatory. Future research should address these limitations by conducting a large-scale, multi-centre, adequately powered, longitudinal, randomised controlled trial in varied geographic and clinical contexts. Such studies should consider systematic assessment of recruitment barriers. Incorporating a health economic analyses would provide valuable insights into the cost-utility of pelvic floor rehabilitation versus traditional care. Additionally, implementation research could investigate the acceptability, scalability, and sustainability of this intervention in routine clinical practice in diverse settings, supporting its wider adoption if proven effective.

## Conclusion

This study demonstrates that structured physiotherapist-led PFR for LARS is feasible and acceptable in an outpatient setting. Exploratory findings suggest potential clinical benefit, providing rationale for future randomised controlled trials to determine the effectiveness of PFR for LARS management within CRC survivorship care.

## Data Availability

The datasets generated during and/or analysed during the current study are available from the corresponding author on reasonable request.
